# Non-antimicrobial interventions in recovery from community-acquired pneumonia in adults

**DOI:** 10.1097/QCO.0000000000001182

**Published:** 2026-01-09

**Authors:** Louise Lansbury, Tricia McKeever, Wei Shen Lim

**Affiliations:** a Faculty of Medicine and Health Sciences, University of Nottingham; b National Institute for Health Research (NIHR) Nottingham Biomedical Research Centre; c Department of Respiratory Medicine, Nottingham University Hospitals NHS Trust, Nottingham, UK

**Keywords:** community-acquired pneumonia, interventions, outcomes, recovery

## Abstract

**Purpose of review:**

We review recent evidence on the effectiveness of non-antimicrobial adjunctive interventions on the recovery of adults diagnosed with community-acquired pneumonia (CAP).

**Recent findings:**

Respiratory physiotherapy, early mobilization or tailored exercises may decrease length of stay (LoS), dyspnoea and readmissions, but there is little evidence of an effect on mortality. Nutritional interventions may decrease readmissions and improve 30-day mortality, but there are few studies on the effect of individual micronutrient supplementation. Strategies to improve discharge communications and patient education may decrease readmission rates, improve treatment compliance and patient satisfaction, whereas the implementation of guidelines and care bundles may decrease 30-day mortality but does not appear to affect length of stay or 30-day readmissions. For adjunctive therapeutic interventions, there is evidence that for severe CAP, corticosteroids probably decrease short-term mortality and possibly longer term mortality and LoS. Antiplatelet agents and statins may decrease short-term mortality.

**Summary:**

A wide range of adjunctive interventions have been trialled aiming to improve patient outcomes with variable results and considerable heterogeneity between studies and populations. Future studies should involve engagement with patient groups to identify uncertainties and outcomes they consider important, utilize a core set of outcome measures, and assess long-term outcomes.

## INTRODUCTION

Globally, pneumonia is a major cause of morbidity and mortality. In 2021, there were an estimated 274 million cases and 1.6 million deaths in adults from lower respiratory tract infections worldwide [1^▪^]. Although mortality rates have declined in past decades, the burden of disease remains substantial, and the absolute number of adults impacted in future years is likely to rise alongside ageing populations [1^▪^].

Appropriate antibiotics remain the mainstay of treatment for pneumonia, and a large body of literature focusses on the role of antimicrobials. Despite an expansion of our understanding of the mechanisms underlying pneumonia and the availability of antimicrobials over the years, the ongoing disease burden indicates that current treatment strategies are suboptimal and that there is a requirement to strengthen the evidence base for additional interventions aimed at optimizing both short-term and long-term pneumonia outcomes on the road to recovery.

In this article, we will consider pneumonia outcomes and how these may be measured, and will then describe the evidence for the effectiveness of nonantimicrobial interventions in treating patients hospitalized with non-COVID-19 community-acquired pneumonia (CAP) and their impact on recovery. 

**Box 1:**
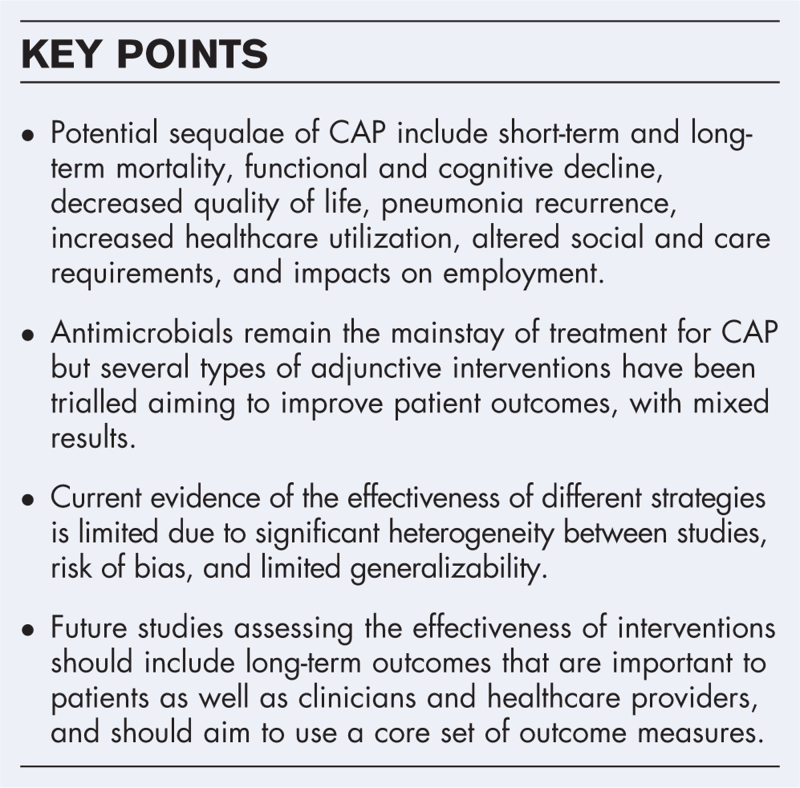
no caption available

## LONG-TERM SEQUELAE OF PNEUMONIA

CAP is associated with adverse outcomes, which may last months or even years after the initial episode and have implications for patients, their families and healthcare resources. A recent prospective cohort study from the USA (PNEUMO) reported that at 6 months after hospitalization with CAP and compared to their prehospitalization status, 12.8% of participants had lost the ability to perform at least one basic activity of daily living (ADL), 22% lost the ability to perform at least one instrumental ADL, 41.6% suffered cognitive impairment, 58.7% had loss of employment and 23.6% had decreased quality of life [2^▪▪^]. Poorer pre-illness ability to perform instrumental ADLs and quality of life, lower education, female sex, former and current tobacco use, past history of dementia and delirium were associated with worsening 6-month outcomes, with the exception of employment [2^▪▪^]. Additionally, patients with CAP have high rates of readmission to hospital, which in turn is associated with significant inpatient mortality [3^▪▪^], high rates of primary care consultation within 7 days of discharge [4], increased risk of cardiovascular events and heart failure [5,6], recurrent pneumonia [7,8^▪^] and persistent symptoms [9]. Every individual's experience of recovery from pneumonia is unique reflecting the complex interplay between their clinical characteristics, existing comorbidities, frailty, physical and mental status, pneumonia illness severity and underlying causative micro-organism.

## MEASURING RECOVERY

Although most people survive an episode of pneumonia and will be discharged into the community to recuperate if hospitalized, clinicians’ and patients’ perception of ‘recovery’ may be discordant; a physician's assessment of clinical cure and resolution of radiological changes may be less important to patients than their own individual perception of their symptoms, functional status and quality of life.

Studies on the management of pneumonia assess heterogenous outcomes but outcomes which are important to patients, health professionals and other stakeholders are often omitted, limiting the comparability and interpretability of results. A recent systematic review of 280 clinical trials of pneumonia management identified 43 distinct outcomes and 108 measurement instruments, with most (97.5%) reporting clinical or physiological outcomes but only 11.8% reporting life impact outcomes with significant variation in the selection of measurement instruments; highlighting the need for development of a rigorous set of core outcomes to be used and reported as a minimum in future trials, thus improving comparability and interpretation [10^▪▪^]. This systematic review was the first step in a body of ongoing work being conducted by the European Respiratory Society Task Force, which includes qualitative interviews with patients recently recovered from pneumonia and a multistakeholder Delphi exercise, the aim of which is to develop a globally representative core outcome set (COS) for pneumonia [11].

## NON-ANTIMICROBIAL INTERVENTIONS AND RECOVERY FROM COMMUNITY-ACQUIRED PNEUMONIA

Evidence from observational studies suggests that potentially modifiable risk factors for poor outcomes in CAP such as mobility, nutrition, frailty and underlying comorbidities may be amenable to interventions that could improve outcomes and promote recovery. However, current guidelines generally focus on the pharmacological management of CAP with few recommendations on adjunctive treatments [11–13]. Table 1 summarizes the evidence for different non-antimicrobial interventions, along with the levels of certainty of the evidence and gaps where further research is required.

**Table 1:** Summary of effectiveness of non-antimicrobial interventions against recovery outcomes in patients with community-acquired pneumonia and gaps where further research is required

Outcomes by intervention	Summary of effectiveness of intervention	Certainty of the evidence^a^	Comment	Research gaps
Physical therapies				
Mortality Readmission Length of stay Dyspnoea	Little or no effect (chest physiotherapy, OMT, chest wall oscillation) [16]. Possible trend to decreased readmission [17^▪▪^]. May decrease LoS (respiratory physiotherapy, early mobilization or tailored exercises)[15^▪^]. May improve dyspnoea levels. [15^▪^].	Low for all outcomes	Lack of large blinded RCTs. Individual studies are mostly small and heterogeneous with short-term outcomes and methodological flaws.	Effectiveness in longer term outcomes, including patient-reported outcomes and cost-effectiveness of physiotherapy. Large adequately powered RCTs. Standardization of physiotherapy methods and standardized outcomes. Patient-reported outcomes.
Nutritional interventions				
Nutritional supplements				
Mortality Readmission Muscle strength ADL	Nonsignificant trend towards reduced 30-day all-cause mortality with individualized nutritional support [24]. Individualized nutrition plan in hospital and postdischarge may decrease readmissions [25]. Improved 6-month grip strength with intervention. No effect on 6-month skeletal muscle index [23^▪^]. No significant effect [23^▪^].	Low for all outcomes.	Lack of large RCTs in patients with CAP. Patients often older and malnourished and differences in nutrient intake at baseline between groups may influence end points.	Large adequately powered RCTs with longer follow-up. Better understanding of the role of micronutrients. Determination of optimal energy intake during hospitalization and postdischarge to help prevent mortality and improve outcomes. Identification of gaps and barriers in the implementation of nutritional support plans.
Vitamin C				
Mortality Readmission Length of stay	Nonsignificant trend towards reduced 28-day mortality [26^▪▪^]. Nonstatistically significant decrease [27^▪▪^]. Nonstatistically significant decrease in LoS [27^▪▪^].	Low for all outcomes	Limited number of small RCTs, methodological flaws and potential biases in included studies.	Large, adequately powered RCTs. Dosage, route of administration, type of preparation, tapering regimen versus abrupt discontinuation. Effects in noncritically ill patients with CAP to prevent clinical deterioration.
Vitamin D				
Chest X-ray resolution	No effect [29].	Low	Only one RCT in adults which failed to meet planned sample size.	Large RCTs in adults, including those with low baseline vitamin D levels. Optimal dosing regimen. Longer term outcomes, including patient-reported outcomes.
Education/communication strategies				
Readmission Patient satisfaction	Decreased readmission of patients with respiratory disease [34]. Higher patient satisfaction 30-days postdischarge [34].	Low for all outcomes	A few small RCTs including respiratory patients but only one specific for CAP and different types of intervention used.	Large RCTs in patients with CAP. Patient comprehension of discharge instructions. Effectiveness of different educational methods for specific patient groups. Impact of technology on postdischarge monitoring and communication. Longer term outcomes and patient-reported outcomes.
Follow-up strategies				
Mortality Readmission	No effect on 30-day mortality [early postdischarge geriatrician and nurse visit to elderly (one quasi-RCT)] [40]. Decreased readmission at 30 days (early postdischarge geriatrician and nurse visit to elderly (one quasi-RCT) [40].	Low	Lack of RCTs.	Recommendations for posthospital discharge care. Longer term outcomes, particularly quality- of life.
Care bundles/guideline implementation				
Mortality Readmission	Possible decrease in 30-day mortality (one stepped-wedge trial) [43]. Little or no effect [43,44].	Low	Potential temporal bias in before and after guideline implementation studies. Results may not be generalizable to different healthcare systems.	Identification of barriers to implementation of guidelines in clinical practice. Impact of guideline implementation on postdischarge and patient-reported outcomes.
Adjunctive therapeutics				
Corticosteroids				
Short-term mortality	Likely decreased 30–60-day mortality [45^▪▪^].	Moderate^b^	There are now several large RCTs including patients with CAP.	More evidence required on type of corticosteroid, dose, duration and which patients would benefit most. Impact of different pathogens on effectiveness of adjunctive corticosteroids.
Long-term mortality	Possible decrease in 90–180-day mortality [45^▪▪^].	Low^b^		
Length of stay	May be reduced [45^▪▪^].	Low^b^		
Mechanical ventilation	Likely decreases progression to invasive mechanical ventilation [45^▪▪^].	High^b^		
Adverse events	May decrease risk of serious adverse events but probably increases risk of hyperglycaemia, and uncertain effect on secondary infections, GI bleeding, neuropsychiatric effects [45^▪▪^].	Low^b^ (serious AEs); Moderate^b^ (hyperglycaemia); Very low^b^ (GI bleeding; secondary infections; neuropsychiatric effects)		
Platelet inhibitors				
Mortality Myocardial infarction Bleeding	Possible reduction in mortality [51^▪▪^]. Little or no effect [51^▪▪^] Uncertain (no events) [51^▪▪^]	Low^c^ Very low^c^ Very low^c^	Short median duration of follow-up in studies. Only two RCTS, mostly observational studies with inherent biases, inconsistent effect estimates and heterogeneity and potential for publication bias.	Large-scale RCTs in patients with CAP. Effectiveness of specific antiplatelet agents. Patient populations most likely to benefit from adjunctive antiplatelet therapy. Optimal timing, dose and duration.
Statins				
Mortality	Possible decreased mortality [52].	Low	Few large RCTS in patients with CAP. Most data from observational studies with small study effects and high heterogeneity.	Large RCTs in patients with CAP. Optimal timing and dose. Specific patient populations who may benefit.

CAP, community-acquired pneumonia; LoS, length of stay; OMT, osteopathic manipulative therapy; RCT, randomized controlled trial.

a Certainty of evidence decision based on number of studies, study design, size, statistical significance, consistency between studies, likely bias.

b Based on GRADE assessment in Pitre *et al.* [45^▪▪^].

c Based on GRADE assessment in Lother *et al.* [51^▪▪^].

## PHYSICAL THERAPIES

Prolonged hospitalization can lead to physical deconditioning due to extended periods of bed rest, decreased mobility and the systemic inflammation caused by the underlying infection, which in turn may result in respiratory muscle weakness, impaired pulmonary function and prolonged recovery [14]. There is much interest in the effectiveness of physical therapies in improving outcomes in patients with CAP. A recent systematic review of RCTs of physical therapies in patients treated for pneumonia in non-ICU settings pooled data from nine studies, reporting that length of stay (LoS) was shorter in patients receiving physical therapy interventions (either respiratory physiotherapy, early mobilization or tailored exercises) [mean difference −1.47, 95% confidence interval (CI) −1.86 to 1.08], and that there was an improvement in dyspnoea levels (mean difference −1.02, 95% CI −1.78 to −0.26) [15^▪^]. However, there was much heterogeneity in the overall results, and lack of data in the included studies did not allow for analysis of longer term outcomes. Regarding mortality, a Cochrane systematic review of chest physiotherapy for pneumonia, which included eight RCTs of five types of chest physiotherapy found little or no effect on improving mortality from either conventional chest physiotherapy, osteopathic manipulative treatment or high-frequency chest wall oscillation; the certainty of the evidence for all types of chest physiotherapy was very low due to limitations of the included studies [16]. A recently published RCT [17^▪▪^] from Denmark evaluating the effect of in-bed cycling or booklet exercises during admission compared to standard care only in 186 patients with CAP found no difference between the groups in terms of LoS or 90-day and 180-day mortality, but did observe a tendency towards a reduced 90-day readmission risk in the exercise group compared to controls (adjusted hazard ratio 0.59, 95% CI 0.33–1.21). Although not powered to show an effect of exercise on readmissions, the lower number of readmissions reported could be clinically and economically relevant.

With the current uncertainties surrounding the effectiveness of physical therapies on longer term outcomes and recovery in pneumonia, more research is required for physical interventions that are delivered in the postdischarge setting, and there are some ongoing studies, which may help to address this. The AMBULATE trial (NCT05725928, due to complete in 2026) is the largest RCT aiming to determine the effects of mobility-technician-assisted ambulation on mobility status and adverse events for elderly medical inpatients (not specifically CAP). Although the sample size of 3000 is powered for the primary outcome of Short Physical Performance Battery score at discharge, the trial should also provide valuable data on the effects of the mobility intervention on postdischarge outcomes, including posthospital functional status. A pilot cohort study aiming to develop a neuromuscular electrical stimulation intervention in patients across in-patients and postdischarge settings to improve cognitive and functional recovery in older adults hospitalized with pneumonia or an acute exacerbation of COPD is currently recruiting in the United States and is due to be completed in late 2025 (NCT05462226). Additionally, a Danish feasibility study (NCT06689280, REHAB-CAI, not yet recruiting) in older persons following a community-acquired infection hospitalization will aim to evaluate standard care versus a patient-centred and individualized exercise intervention that is kick-started in hospital and continued for 3 months after discharge with video-supervised home exercises.

## NUTRITIONAL OR SUPPLEMENT INTERVENTIONS

Malnutrition, defined as a lack of intake or uptake of nutrition leading to altered body composition and body cell mass leading to diminished physical and mental function and impaired clinical outcome from disease, may be a consequence of starvation, disease and old age either alone or in combination [18]. It has been estimated that up to 53% of elderly patients hospitalized with CAP are malnourished, although estimates vary depending upon the population and definitions used [19,20], and that undernourished patients with CAP have a higher risk of 90-day mortality compared to those who are well nourished [odds ratio (OR) 3, 95% CI 1.0–21.4] [21]. Poor nutritional and functional status are likely part of a complex relationship between the causes and consequences of CAP. A recent prospective cohort study of 144 patients hospitalized with CAP observed that after an average 44.6 days post diagnosis, 28% of community-dwelling patients and 67.9% of those institutionalized were malnourished as indicated by the Mini-Nutritional Assessment (MNA), with the risk of malnutrition being 44 and 9.5%, respectively. Furthermore, micronutrient deficiency was highly prevalent with zinc (61.8%), vitamin D (54.4%) and vitamin C (45.1%) deficiencies most common [22^▪▪^]. Although protein and micronutrient deficiencies are potentially modifiable, few studies have addressed interventions in this area in adults.

### Nutritional supplementation

Three recent RCTs have investigated the effect of nutritional interventions on outcomes in CAP. A Japanese RCT, which aimed to investigate the effects of increased dietary protein for 6 months on maintaining physical function and ADL in 169 older hospitalized adults at least 75 years deemed to be at nutritional risk (scoring <11 on the MNA-Short Form), reported that compared to standard care, grip strength improved in the intervention group from baseline (between-group difference 1.29 kg, 95% CI 0.22–2.36, *P* < 0.05), and although skeletal muscle index and leg strength improved in both groups and was greater in the intervention group, the difference between the groups did not differ significantly, and there were no significant improvement in ADL indices [23^▪^]. However, only 27% of enrolled participants had a primary diagnosis of CAP, and there were baseline differences in nutrient intake at baseline between the intervention and control groups, which may have influenced the endpoints.

In another study, a secondary analysis of data from 378 participants with confirmed lower respiratory tract infection who were enrolled in a RCT (EFFORT) evaluating the effect of individualized nutritional support to reach energy and protein goals compared to standard hospital food, reported a 25% reduction in the risk of 30-day all-cause mortality (12.2 versus 9.1%), which although not statistically significant in itself was similar to the significant mortality benefits observed in the overall EFFORT cohort (OR 0.65, 95% CI 0.47–0.91) [24].

Finally, a RCT of an individualized nutritional intervention program in 82 malnourished older adults with pneumonia, which included postdischarge dietician monitoring and prescription of individualized plans in the intervention group, observed that nutritional status improved and readmission rates declined (OR 0.23, 95% CI 0.06–0.87) in participants who received the intervention [25]. The benefits on nutritional status were not observed during the index hospitalization, highlighting the importance of including longer term assessment of outcomes in trials evaluating recovery.

### Vitamin C

A recent systematic review and meta-analysis of data from six RCTS on the efficacy of vitamin C for treatment of CAP, reported an overall nonsignificant trend towards reduced mortality in the vitamin C group compared to controls (excluding COVID-19 studies RR 0.46, 95% CI 0.13–1.62, *I*^2^ = 0%, three studies) [26^▪▪^]. The most recent RCT included in the systematic review, which included patients with moderate to severe CAP (CURB-65 ≥2), did not find any significant difference in 28-day mortality, LoS, time to clinical stability or 30-day readmission between the group treated with adjunctive intravenous then oral vitamin C for 7 days [27^▪▪^]. This was, however, a relatively small feasibility study in which only 75 patients were randomized, and a much larger sample size of 932 would be required to detect a risk ratio of 0.5 assuming mortality in the control group of 10%. Similarly, the effect of vitamin C on quality of life or specific CAP-related symptoms after discharge would likely be small and would require a very large multicentre study.

### Vitamin D

Most studies of vitamin D supplementation as an adjunctive treatment for pneumonia have been conducted in children. A systematic review of RCTs [28] found only one relevant study in adults [29] in which 117 participants with CAP were randomized to receive either a single oral dose of vitamin D_3_ (200 000 IU) or placebo. No effect on the primary outcome of complete resolution of chest radiograph infiltrate at 6 weeks posttreatment was found, although the trial was stopped early due to recruitment difficulties arising from the large number of potential participants who were ineligible because of prior vitamin D consumption, or unable to give consent.

## EDUCATIONAL AND COMMUNICATION INTERVENTIONS

Limitations in patients’ ability to recall information about their condition and its management at discharge, whether due to cognitive impairment, anxiety, poor health literacy, or language barriers, may result in treatment failure and hamper their recovery postdischarge, and have been associated with a higher risk of mortality and readmission [30–33].

Discharge communication and patient education about their management is a potentially modifiable factor, which may lead to improvements in outcomes and quality of care. A systematic review of 60 RCTs of discharge communication interventions found lower readmission rates in the intervention group compared to controls risk ratio (RR) 0.69, 95% CI 0.56–0.84), higher adherence to the treatment regimen (RR 1.24, 95% CI 1.13–1.37), and higher patient satisfaction (RR 1.41, 95% CI 1.20–1.66) [34]. However, few studies have specifically looked at these interventions in CAP patients. The largest is the EDUCAP trial [35]; the intervention, an educational programme delivered twice predischarge with advice on fluid intake, medication adherence, vaccination and disease knowledge and management, together with a CAP self-management handout, was associated with significantly lower 30-day ED reattendance in the intervention group (11%) compared to controls (26%, *P* = 0.007), fewer hospital re-admissions (5 versus 17%, *P* = 0.007), greater patient satisfaction (82 versus 18%, *P* < 0.001), and better knowledge about their diagnosis (98 versus 20%, *P* < 0.001), although there was no significant difference between the groups in terms of medication adherence [35]. It should be noted that the group who received only conventional information tended to be older and with lower educational levels than the intervention group, and long-term mortality was not assessed.

## FOLLOW-UP STRATEGIES

British Thoracic Society guidelines (updated 2015) recommend clinical review of all patients at around 6 weeks with either their general practitioner or in a hospital clinic [36], but otherwise current guidance on follow-up care after pneumonia is sparse. The American Thoracic Society guidelines conditionally recommend that routine follow-up chest imaging is not indicated in those with symptom resolution within 5–7 days [37^▪▪^,^38^,^39^^▪▪^]. A quasi-RCT of 1330 patients aged at least 75 years reported that patients receiving the intervention of a geriatrician and nurse home visit in the first few days after discharge were less likely to be readmitted within 30 days compared to controls who only received follow-up visits at the discretion of a community nurse or GP (12 versus 23%, aHR 0.50, 95% CI 0.38–0.65, *P* < 0.001), although only 19% of the included patients had pneumonia [40]. No effect was seen on 30-day mortality, but the study was not powered for this outcome and a larger sample size would be required to detect an effect on this outcome.

A recent study from the United States in which 20 primary care clinicians working in diverse settings were interviewed to identify facilitators and barriers to the delivery of postdischarge care, identified at least seven core elements common in follow-up care after severe pneumonia; safety assessment, medication management, medical specialty follow-up, integrating the hospitalization into the primary care relationship, assessing mental and physical well being, rehabilitation follow-up, and social context of recovery [41^▪^]. However, although the study provided expert insight into optimal follow-up practices, the objective effectiveness of these practices was not assessed, and further research is required to inform best follow-up practice in patients recovering from pneumonia.

## CARE BUNDLES AND GUIDELINE ADHERENCE STRATEGIES

Well established guidelines can help clinicians diagnose and treat patients with pneumonia, although they are often underused due to clinicians’ lack of awareness or familiarity with the guidelines, personal attitudes and lack of agreement with the recommendations [42]. Strategies to improve adherence include dissemination, education and training, social interaction, decision support systems and standing orders [42]. Studies of interventions to implement care bundles or guidelines have largely focused on reducing 30-day readmissions, LoS and mortality, with varying results in these areas. A recent stepped-wedge, cluster-controlled trial across 16 community hospitals in the United States observed that deployment of an open-loop clinical decision support embedded within the electronic health record was associated with improved processes of care (time to first antibiotic and outpatient disposition) and lower 30-day all-cause mortality in patients treated in the emergency department for pneumonia (aOR 0.62, 95% CI 049–0.79), although readmission rates did not change [43]. A UK study, which examined outcomes in 16 201 patients from eight acute hospitals following implementation of a quality-care bundle consisting of seven British Thoracic Society guidance-based CAP measures, observed a decrease in crude mortality over the 3-year study period, although LoS and readmission rates did not change despite improvements in compliance (albeit suboptimal) with the care bundle. Interestingly, mortality rates were higher in those patients who passed the individual bundle measures (except the CURB-65 recording measure) compared to those who did not, suggesting that those with more severe CAP might be recognized and the care bundle more likely to be implemented in the management of these patients than those with less severe disease [44].

## ADJUNCTIVE NONANTIMICROBIAL THERAPEUTIC INTERVENTIONS

Antimicrobials are the mainstay treatment for CAP, but there has been much interest in adjunctive treatment with other therapeutic agents based on their anti-inflammatory properties and potential to attenuate the systemic inflammatory response.

### Corticosteroids

A recent systematic review and meta-analysis of 30 RCTs in adult CAP patients reported that corticosteroids probably reduce short-term mortality (up to 60 days) (RR 0.82, 95% CI 0.74–0.91) with moderate certainty, although the effect was only significant in the subgroup with more severe CAP, and may reduce long-term mortality (RR 0.89, 95% CI 0.76–1.03) [45^▪▪^]. The requirement for mechanical ventilation may also be decreased (RR 0.63, 95% CI 0.48–0.82), and LoS may be shortened (mean difference 2.30 days, 95% CI 0.81–3.81 days or fewer). However, there is probably an increase in the risk of hyperglycaemia requiring intervention (RR 1.32, 95% CI 1.12–1.56) and the impact on gastrointestinal bleeding, secondary infections and neuropsychiatric effects remains uncertain [45^▪▪^]. Included in the systematic review were the results from the most recent RCTs, including new data from the REMAP-CAP trial [46^▪▪^], the ESCAPe trial [47], CAPE-COD [48^▪▪^], APROCCHSS [49^▪▪^] and the STEP trial [50^▪▪^]. Despite the relatively large number of trials that have been conducted in this area, questions remain regarding the type, dose and route of administration of corticosteroids that are most effective, and their effectiveness according to the underlying pathogen.

### Antiplatelet agents

There is some low-certainty evidence that antiplatelet agents may be associated with reduced mortality compared with usual care or placebo from pooled analysis of 13 observational studies and two RCTs (hazard ratio from observational studies 0.65, 95% CI 0.46–0.91, aOR from observational studies 0.67, 95% CI 0.45–1.00) and RR from RCTs (0.66, 95% CI 0.25–2.25) [51^▪▪^]. Although one included RCT reported a significant reduction in the incidence of myocardial infarction (RR 0.10, 95% CI 0.01–0.79), no benefit was seen when observational data were pooled (RR 6.69, 95% CI 0.16–271.6). Overall, median duration of the studies was short, and as the risk of cardiovascular outcomes is increased for weeks to months postdischarge, it is possible that outcomes measured longer term may be different.

A large RCT to evaluate the effectiveness of aspirin compared to standard care without aspirin to reduce the risk of major adverse cardiovascular events up to 90 days in patients at least 50 years hospitalized with CAP is currently ongoing and due to end in 2027 (ASPECT https://www.isrctn.com/ISRCTN85630652).

### Statins

Although there has been interest in the use of statins (3-hydroxy-3-methyl-glutaryl-coenzyme-A reductase inhibitors) to improve the immune response to infections, studies have shown discordant results. An umbrella review of meta-analyses, which included separate re-analysis of pneumonia studies, noted that the risk of mortality was significantly decreased in four of the six meta-analyses (all for observational studies) reporting adjusted odds ratios, ranging from 0.59 (95% CI 0.48–0.73) to 0.71 (95% CI 0.64–0.79), with the level of evidence judged to be ‘weak’ for all of them based on small study effects or high between-study heterogeneity [52]. It is likely that the patient population, dose, type and timing of statins will impact outcomes in CAP. A pilot RCT of 4 days of high-dose simvastatin in 62 patients at least 55 years with CAP plus sepsis not admitted to ICU found improvements in systemic neutrophil function and a reduction in systemic neutrophil elastase burden and improved Sequential Organ Failure Assessment scores compared to placebo, with increased hospitalization free survival at both 180 and 365 days [odds ratio (OR) 0.45, 95% CI 0.22–0.93; and OR 0.45, 95% CI 0.22–0.90 respectively] [53]. High-dose simvastatin was well tolerated in this study, but it was not powered for the clinical endpoints, suggesting the need for a definitive large multicentre trial to clarify the efficacy of statins on mortality and readmission and other outcomes considered to be important by patients themselves.

## CONCLUSION

Appropriate antimicrobial treatment is the mainstay treatment for CAP and current pneumonia guidelines focus largely on this, but a wide range of adjunctive treatment strategies have been trialled in efforts to decrease the substantial mortality and morbidity burden associated with the disease. Evidence for the effectiveness of such strategies in preventing the adverse sequalae of CAP during hospitalization and during the recovery period is largely weak, with mixed results, residual confounding in observational studies, high risk of bias, and considerable heterogeneity between studies in terms of their design, outcome measurements, and included population. The ongoing development of a set of core outcome measures for use in future trials in this area should help to alleviate some of these discrepancies.

Engagement with patients with experience of CAP will be essential, through exercises such as the ongoing James Lind Alliance CAP Priority Setting Partnership in the UK, in order to identify uncertainties about the diagnosis, complications and management of pneumonia and to ensure that the outcomes that patients consider to be important are incorporated into the design of future studies.

## Acknowledgements

*None*.

### Financial support and sponsorship

*The National Institute for Health Research (NIHR) Nottingham Biomedical Research Centre, UK, provided salary support for this work.*

### Conflicts of interest

*There are no conflicts of interest.*

## REFERENCES AND RECOMMENDED READING

Papers of particular interest, published within the annual period of review, have been highlighted as:

▪ of special interest
▪▪ of outstanding interest
